# Dental restoration selection by students at southern Saudi Arabian universities

**DOI:** 10.6026/97320630018986

**Published:** 2022-10-31

**Authors:** Waleed Asiri, Siraj DAA Khan, Mohammad Abdulkareem, Hamood Abdullah, Farhan Salah, Khalaf Nasseri, Fahad Fayez

**Affiliations:** 1 Faculty of Dentistry, Najran University, KSA

**Keywords:** Amalgam, composite, ceramic, indirect restoration, direct restoration

## Abstract

Dental caries is a serious oral health concern and restoration of teeth is the main solution to this issue. Various restorative materials having different properties are used for posterior restoration. The aim of this study was to find that which factors can affect the choice of selection of material for posterior restoration among dental graduate students. For this purpose, a questionnaire was shared electronically among students of three different universities (King Khalid University, Jazan University, and Najran University). After collection, data were subjected to a chi-square test to check the significance. It has been observed that almost 50% of participants choose composite resin for posterior restoration. Results showed that according to most (more than 50%) of the dental students in Southern Saudi Arabia, different patient and tooth condition related factors can affect the choice for selection of restorative material.

## Background:

Dental caries is considered a public health issue for both permanent and primary teeth, it shows a high rate of prevalence and it affects 621 million children and 2.4 billion adults globally. [1] Modern technique to solve this problem involves the diagnosis and then determines the patient's status for caries risk, followed by the different intervention strategies to prevent arrest and reverse the process of caries and delay the treatment of restoration until it becomes necessary. [2] When the tooth structure is damaged permanently, the cleaning and filling of the cavity with restorative material is the commonly used technique. [3] This therapy has many advantages as it restores and preserves the structure of the tooth as well as dental pulp. [4] The longevity of restoration is affected not only by the selected material but also by other factors such as characteristics of patients, the ability of operators and the isolation method used. [5] Consequently, materials that have adhesive properties have been largely used, they are easy in handling and meet the esthetic demands of patients. [6] The available options as restorative materials are amalgam, cement, composite resin, gold, compomer, ceramic and metal alloys, depending on the preference of patients and defect size. All these materials vary in physical, esthetic, biological and cost aspects. Amalgam is simple in processing, can bear moisture contamination, presents excellent durability and leaves a less marginal gap that minimizes the caries risk. [7] On the other hand, composite resin provides acceptable clinical performances and high survival rates in long-term follow-up, that's why it is considered as an appropriate direct restorative material both in primary and permanent dentitions in posterior as well as in anterior teeth. [8, 9] The disadvantages are that it is expensive, prone to fracture and brittle. It has been shown through many clinical studies that composite resin hasa satisfactory longevity rate. [10] For premolar and molar cavities restoration, resin composite and amalgam are still the best selection. The proffered choice of amalgam as a restorative material for posterior teeth has now been replaced with resin composite. However, retrospective studies and surveys vary in their conclusion about the most commonly used material in dentistry nowadays. [11] The literature has pointed out, though, that patient-related risk factors, such as the presence of high stress and/or elevated caries risk, have an important influence on restoration longevity. Therefore, the primary objective of this survey is to find that which factors can influence the choice of dental students for the selection of restorative material.

## Material and Methods:

A questionnaire was distributed among graduate dental students from three different universities (King Khalid University, Jazan University, and Najran University) of Southern Saudi Arabia. They received questionnaires electronically through email and WhatsApp. The purpose of this survey was explained to the students. The students who are directly involved in dental care and gave their consent were included in this study. Those who did not agree and didn't give consent were excluded. Total 17 questions were asked in the survey about the selection of restoration material for posterior teeth and which factors can affect the selection of restoration material. Total 260 students participated; the response of participants was summarized in tables. Detailed response of students was entered in an Excel spreadsheet. Complete data were collected, arranged and compiled, arranged systematically and analyzed in terms of percentage frequencies. A Chi-square test was applied to check the significance of data using SPSS-21.

## Results:

A total of 260 students participated in this survey. Out of them, 150 were from King Khalid University, 37 from Jazan University and 73 students from Najran University. About the preference of restorative material 130 (50%) participants out of 260 chose composite resin restoration and 63 (24.2%) selected amalgam restoration. 68.8% and 69.2% of respondents said that they will select composite resin restoration for male and female patients respectively. If the patient is uncooperative, 103 (39.6%) participants out of 260 will choose composite restoration, 44 (16.9%) will select amalgam restoration, 76 (29.2%) will use indirect restoration and according to 37 (14.2%) participants, there is no difference between materials for an uncooperative patient.89 (34.2%) students said that they will select composite resin for the patients with a high risk of caries. 59 (22.7%) participants will choose amalgam restoration for those patients. 68 (26.2%) will select ceramic and 44 (16.9%) will select Gold restoration. All responses showed significant results statistically (P<0.01) except the question about selection for an uncooperative patient (Table 1 - see PDF). According to 192 participants (73.8%) said that patient's desire could affect the selection of restoration material. According to 179 (68.8%) students, patients' age can affect the selection of restoration material while 28 (10.8%) thought that age does not affect the selection. Tooth position and technique sensitivity can affect the selection type according to 68.5% and 69.6% of participants respectively while 12.7% and 12.3% of students don't have knowledge about it. 66.5% of respondents agreed that the cost of restoration is an important factor for the selection of restoration material and 10.4% said that cost does not affect the selection. The extension of caries also affects the selection of material according to 171 (65.8%) participants. The result is statistically significant (P<0.01) (Table 2 - see PDF). According to112 (43.1%) participants among 260 thought that it is possible to put composite resin restoration over amalgam restoration to avoid galvanism while 83 (31.9%) said that it is not possible. About amalgam restoration 58 (22.3%) participants were agreed that this restoration is better with bonding and 66 (25.4%) thought that it is better without restoration while most of the participants (36.2%) thought that both types of amalgam restoration are the same in retention. For destroyed tooth amalgam restoration is better according to 71 (27.3%) students while 49 (18.8%) participants agreed for composite restoration. 97 and 43 respondents said that GIC and indirect restoration is better for this type of tooth respectively. 149 (57.3%) said that they always ask the patients if they had sensitivity towards any material. Amalgam restoration and composite restoration has the best longevity according to 122 (46.9%) and 94 (36.2%) participants respectively while 30 (11.5%) students don't have knowledge about it. All responses were significant statistically (P<0.01) (Table 3 - see PDF).

## Discussion:

Over the past 25 years, surveys on the international level reported an increase in the clinical experiments and teaching of posterior composite restorations. [12] In the present study, composite resin was the choice for 50% of the participants. For an increase in longevity, many dentists choose tooth-coloured restorations such as composite resin restorations. [13] Another study also reported that composite resin was the best choice to treat the posterior teeth of patients. [14] It has been reported that most of the cavities are restored by using composite resin, however, 30% were restored with amalgam. [15] Similar results were reported in 2011, where 42% of dentists in Israel used composite posterior restoration. [16] In the USA, the composite resin was used by 50% of dentists. [3] This is because most of the dentists were young and they follow the new trend. [17] The use of composite as posterior restoration was much higher than amalgam among the dentists of Oceania (64% vs 19.5%). In the same study, variations are also present among the students of different schools, some do not use amalgam restoration at all and other schools use 50% amalgam and 50% composite restoration.[18] Previous studies in different countries reported the use of composite resin restoration and amalgam restoration e.g., In Japan (2009) 45% of dentists used composite restoration and 0% used amalgam; [19] In Ireland and United Kingdom(2010) 44% of dentists used amalgam and 55% used composite resin restoration; [20] In Canada and USA(2011) 48% used amalgam while 49% used posterior composite restoration [21] and in Spain (2012) 26% of dentists used posterior amalgam restoration and 44% used composite restoration. [22] In the current survey 89% of students said in case of high risk of caries, they will use composite resin restoration while 59% will use amalgam restoration for patients who have a high risk of dental caries. This choice seems consistent with evidence that there is a major difference in the selection of restorative material for high and low risk of caries. Patients with a high risk of caries were negatively influenced by the use of composite restoration [23] and it has been reported that composite resin had more chances of occurrence of dental caries as compared to amalgam. [24] The limitations of this study were that this study is carried out for specified universities, and the students who participated in the survey need a sufficient amount of effort and time to answer the questions.

## Conclusions:

It has been concluded that different factors such as patient's gender and age, tooth position, cost of restoration and caries extension may affect the selection of restoration material for posterior restoration. However, it mainly depends upon the dentist's knowledge about the different materials. Composite resin restoration was the best choice for most of the participants in different situations but amalgam was also the choice for many dentists. There is a need to improve the knowledge of dental graduates in terms of restoration material, their longevity and other factors through teaching and clinical experiments.
